# Management of locally advanced laryngeal cancer

**DOI:** 10.1186/1916-0216-43-4

**Published:** 2014-01-28

**Authors:** Alexander D Karatzanis, Georgios Psychogios, Frank Waldfahrer, Markus Kapsreiter, Johannes Zenk, George A Velegrakis, Heinrich Iro

**Affiliations:** 1 Department of Otorhinolaryngology, Head and Neck Surgery, University of Erlangen-Nuremberg Medical School, Erlangen, Germany; 2 Department of Otorhinolaryngology, Head and Neck Surgery, University of Crete Medical School, Heraklion, Crete, Greece

**Keywords:** Larynx, Advanced carcinoma, Survival, Local control, Treatment, Prognosis

## Abstract

**Background:**

Management of advanced laryngeal cancer is complex and ideal strategy is yet to be defined. This study evaluates the experience of a single head and neck oncologic centre in the management of T4 laryngeal cancer.

**Methods:**

Retrospective assessment of cases primarily treated for T4a squamous cell carcinoma of the larynx, between 1980 and 2007, at a tertiary referral center.

**Results:**

A total of 384 cases were studied. Five-year disease specific survival was 56.2% and local control 87.4%. Regional and distal control estimates were 90.3% and 88.3% respectively. Prognosis was significantly superior for cases treated with primary surgery compared to cases solely managed with non-surgical modalities. Positive surgical margins and regional disease worsened prognosis.

**Conclusion:**

This study suggests that primary surgery remains a key element in the treatment of advanced laryngeal cancer. The need for well-designed, prospective, randomised studies in order to further evaluate the remaining role of primary surgery in the modern management of locally advanced laryngeal lesions is emphasized.

## Introduction

Laryngeal cancer represents one of the most common head and neck malignancies, accounting approximately for 20% of all cases. The vast majority of tumors are squamous cell carcinomas [1,2]. Up to 40% of patients present with advanced disease [3]. Due to the important physiologic functions of the larynx, advanced laryngeal lesions are associated with significant morbidity and mortality for the patient and increased financial costs for society [4,5].

Management of advanced laryngeal cancer is complex and the ideal strategy is yet to be defined [6]. Treatment has so far included total laryngectomy (TL), alone or with neck dissection (ND), radiotherapy (RT) alone, TL followed by RT, and combined chemotherapy and RT (CRT) [6,7]. TL followed by adjuvant RT has been widely considered the standard management option for many years [8]. However, a shift toward organ-preservation strategies with the use of primary CRT has been recently noted [8,9].

In order to define the ideal management, different aspects must be considered. These include oncologic outcomes, functional results and morbidity, as well as financial costs. Currently, a lack of large-scale prospective studies comparing different management options for advanced laryngeal cancer is noted. In this context, non-randomized data may offer some basis for treatment decision-making. This study aims to evaluate the experience of a single head and neck oncology center in the management of T4 laryngeal cancer.

## Methods

A retrospective study was conducted at an academic tertiary referral center (Department of Otorhinolaryngology, Head and Neck Surgery, University of Erlangen-Nuremberg Medical School, Erlangen, Germany). Relevant approval from the institutional review board of the hospital was obtained. The files of all patients primarily treated for T4a category carcinoma of the larynx, between 1980 and 2007, were evaluated. Patients with recurrent or systemic disease at the time of diagnosis, and histology other than squamous cell carcinoma, as well as patients with second primary tumors at the time of diagnosis, were excluded from the study.

All pathology reports were reviewed and staging was conducted in accord with the 2010 American Joint Committee on Cancer (AJCC) and Union Internationale Centre Contre Cancer (UICC) classification [10]. T4a cases of laryngeal cancer include any supraglottic, glottic, or subglottic lesions that invade through the thyroid cartilage, or invade tissues beyond the larynx, e.g., trachea, soft tissues of neck including deep/extrinsic muscle of tongue (genioglossus, hyoglossus, palatoglossus, and styloglossus), strap muscles, thyroid, oesophagus. Tumors invading prevertebral space, or mediastinal structures, or encase carotid artery, are considered T4b and were therefore excluded from this study. Since T4 carcinomas had been subdivided into T4a and T4b in 2002, the files of patients with T4 tumors treated prior to this date were carefully re-assessed to differentiate between T4a and T4b. Standard diagnostic investigations reviewed included ultrasonography and computed tomography. Magnetic resonance imaging was also used in a few cases. The appropriate treatment modality had been decided by the interdisciplinary tumor board in every case. Factors that mainly influenced the decision included the operability of the tumor, general health status and personal preference of each patient.

All patients were assessed for Disease Specific Survival (DSS) and Overall Survival (OS) as well as Local Control (LC) rates, with respect to T classification, N classification, type of primary treatment, status of surgical margins, and adjuvant therapy. Surgical margins were evaluated from primary tumor pathology reports and considered as positive when characterized by the presence of invasive carcinoma at the edge of resection on permanent section pathology.

Five year DSS was defined using the time from the date of diagnosis to death from the tumor or complications of treatment. Time to LC or regional control (RC) was calculated from the date of initial diagnosis to the date of most recent clinical review when local or regional recurrence was confirmed. Local recurrence was defined as invasive carcinoma developing after completion of initial treatment at the anatomic site of the primary tumor. Regional and distal recurrences were defined as the presence of the same tumor in the regional lymph nodes or distant sites respectively, after the completion of initial treatment. Calculations of five-year overall and disease-specific survival, local control and regional control were made with Kaplan-Meier estimates and compared by the means of the log-rank test. A *p* value of less than 0.05 was considered significant. Statistical analysis was performed using SPSS Version 19 (SPSS In., Chicago IL, USA).

Cases managed with surgery were additionally evaluated for incidence of major complications. Unfortunately, no data regarding complications of non-surgical modalities were available for assessment. Major surgical complications were defined as those which necessitated prolonged hospitalization, blood transfusion, additional surgery, or admission to the intensive care unit. Pharyngeal function was indirectly evaluated by assessing the incidence of permanent gastrostomies.

## Results

A total of 384 cases that satisfied the inclusion criteria were analyzed. Among these, 354 were men and 30 women, approximating a 12:1 men to women ratio. Mean age was 59 years, ranging from 31 to 91 years. Mean follow-up period was 4.7 years (median 2.199, range 0.2-26.1). When classified in accord with anatomic location, 208 cases (54.1%) were supraglottic carcinomas, 142 cases (36.9%) were glottic, and 15 cases (4%) were subglottic; 19 additional cases (5%) could not be further classified. In accord with pathology, 258 cases (67.1%) were classified as well differentiated (grade I or II) and 103 cases (26.8%) as poorly differentiated (grade III or IV). A detailed description of demographics, tumor localization, N status, and histological differentiation, is presented in Table 1.

**Table 1:** Detailed description of demographics, tumor localization, histological differentiation, and N status of all cases in this series

**Parameter**	**Characteristic**	**Total number (cases)**	**Relative frequency (%)**
Sex	Male	354	92.2
	Female	30	7.8
Age (grouped)	≤59	194	50.5
	>59	190	49.5
Smoking status	Smokers	264	68.8
	Ex-smokers	81	21.1
	Non-smokers	39	10.1
	n.a.	384	
Tumor localization	Supraglottic	208	54.2
	Glottic	142	37.0
	Subglottic	15	3.9
	Not specified	19	4.9
Histological differentiation	G1	26	6.8
	G2	232	60.4
	G1/2	258	67.2
	G3	88	22.9
	G4	15	3.9
	G3/4	103	26.8
	Unknown	23	6.0
Total N	N0	188	49.0
	N1	29	7.6
	N2a	7	1.8
	N2b	50	13.0
	N2c	75	19.5
	N3	35	9.1

**Figure 1:**
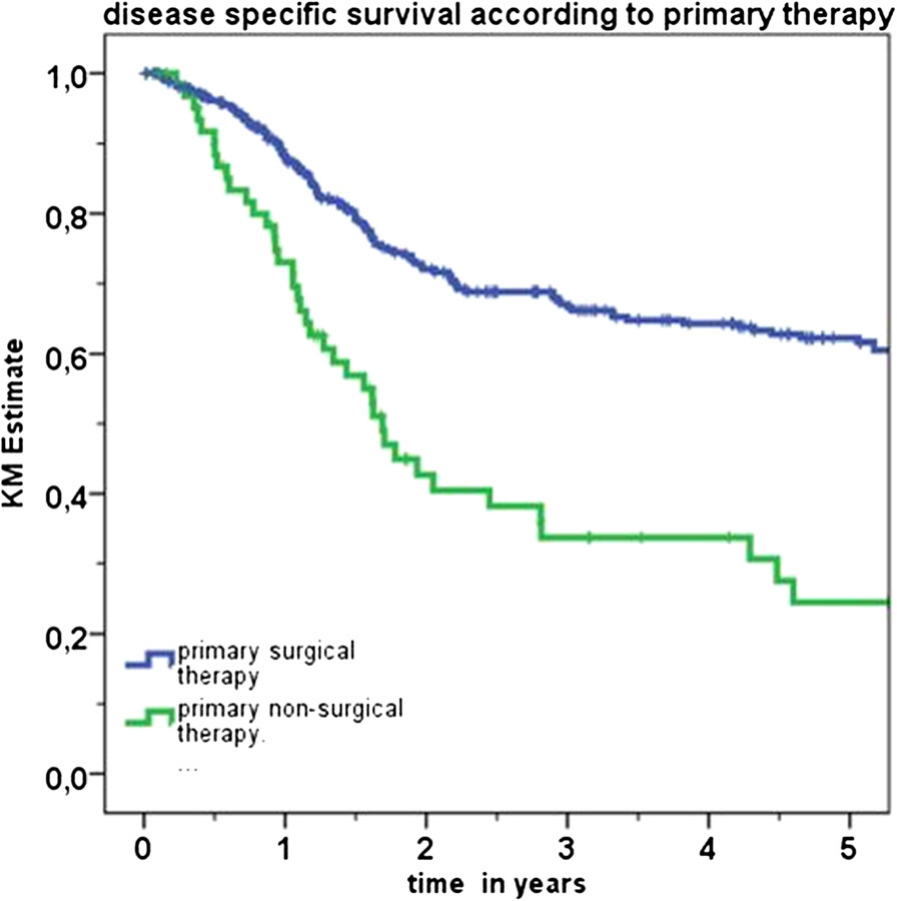
Kaplan-Meier analysis of disease specific survival (DSS) in accord with primary treatment.

Five-year DSS was 56.2% overall in this series while LC was 87.4%. Regional and distal control estimates were 90.3% and 88.3% respectively. Two major groups could be defined in accord with management. One received radiotherapy with or without chemotherapy as primary treatment (CRT group) and if needed salvage surgery (63 cases). The other underwent primary surgery (321 cases) with or without adjuvant CRT. Table 2 shows the detailed treatment variation in the two groups. Although cases were unevenly distributed among the different forms of treatment, prognosis was found to differ significantly between the two groups. Patients treated with surgery and CRT were found to have superior prognosis. DSS rate was 62.2% for the primary surgery group and 24.5% for the CRT group (p < 0.001). OS rates were 41.1% and 16.7% (p < 0.001) respectively. Kaplan-Meier analysis of DSS in accord with primary treatment is presented in Figure 1. Moreover, OS rates were 41.1% for the primary surgery group and 16.7% for the CRT group (p < 0.001). On the other hand, comparable results were found with regard to LC as the former group achieved 87.6% and the latter 83.6% rates (p not interpretable).

**Table 2:** Oncologic results in accord with management strategy

**Therapy**	**Number of cases**	**DSS (%)**	**OS (%)**	**LC (%)**
**OP**	88	53.9	31.1	81.7
**OP + RT**	199	62.6	42.0	88.8
**OP + RCT**	34	80.8*	64.3	93.5*
**RT (+/- salvage surgery)**	35	21.5	11.7	73.9*
**RCT (+/- salvage surgery)**	28	28.8	23.1	94.7*
**Total**	384	56.2	37.2	87.4

OP: primary surgery only. OP + RCT: primary surgery plus adjuvant radiotherapy. OP + RCT: primary surgery plus adjuvant chemoradiation. RT: primary radiotherapy. RCT: primary radiochemotherapy. DSS: disease specific survival. OS: overall survival. LC: local control.

*low number of cases.

Decision to perform surgery as primary treatment was mainly based on local extent of disease, and general health status as well as personal preference of each patient. TL with some form of ND according to the status of the neck was the surgical procedure typically performed. The majority of cases undergoing primary surgery (233/321) also received adjuvant treatment consisting of radiotherapy with or without chemotherapy. However, for reasons that are difficult to detect retrospectively, 88 cases were spared from adjuvant treatment. Usual causes included refusal of the patient, comorbitities, and death before application of adjuvant therapy. Interestingly, oncologic results among these two surgical subgroups were not found to differ significantly. It is emphasized again that comparison is hindered by the unequal distribution of cases among the two subgroups. DSS and LC were 64.3% and 89.5% respectively for the first subgroup, and 53.9% and 81.7% respectively for the second subgroup (p = 0.074 for both DSS and LC).

**Figure 2:**
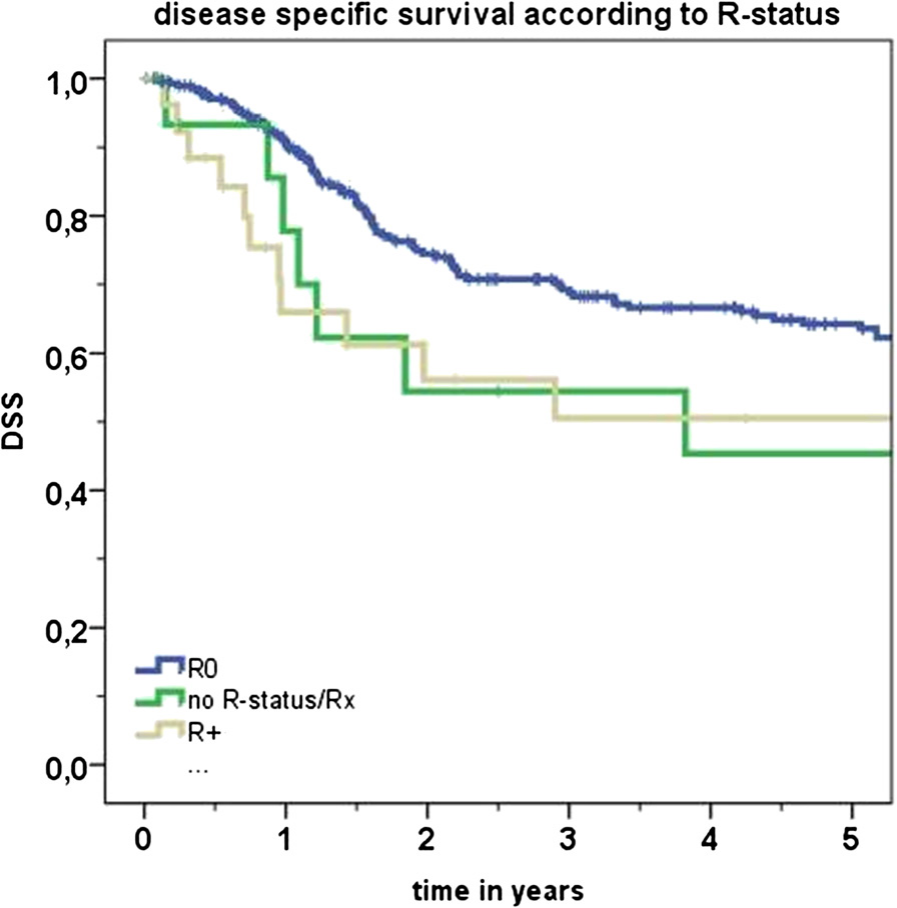
**Kaplan-Meier analysis of disease specific survival (DSS) in accord with status of surgical margins (R).** R0: negative surgical margins. R+: positive surgical margins.

According to pathology reports, negative surgical margins (R0 status) had been achieved in 278 out of 321 (86.6%) surgically treated cases in this series. Conversely, 27 (8.4%) cases had positive surgical margins (R + status) at the end of surgical treatment. All of these cases later received adjuvant treatment. For an additional 16 cases R status could not be determined. Survival rates were found to be superior for cases with R0 status compared to R + status cases (DSS 64.2% versus 50.0% respectively). However, R + status group was comparatively very small thus limiting the statistical power of the log-rank test. Kaplan-Meier analysis of DSS according to R status is presented in Figure 2.

Patients that were not treated with primary surgery received radiotherapy with or without chemotherapy. Selection of the exact treatment scheme was individualized according mainly to the extent of disease and the general health status of each patient. Non-surgical treatment has been affected by various changes in protocols as well as technical developments that have been noted over the years in this centre. For relatively recent cases, however, non-surgical treatment has typically comprised of radiation therapy with a cumulative dose of 70-72 Gy (mean dose 60.71, median dose 60.7, range 26-80 Gy) using conventional fractionation, plus concomitant cisplatinum-based chemotherapy. Salvage operation was typically performed 8-12 weeks following completion of CRT in cases where residual disease had been identified.

Clinical or histological evidence of regional disease was found in 196 (51%) out of 384 cases at the time of initial management. A detailed presentation of N status may be found in Table 1. The presence of regional metastases affected prognosis. DSS rates were 66% for N0 and 46.2% for N + cases (p = 0.002). Similarly, OS rates were 44.9% and 29.5% respectively (p = 0.001). In all cN0 cases undergoing surgery, bilateral selective dissection of levels II, III, and IV was performed. From 144 cN0 cases, 116 underwent elective ND and 35 proved to be pN+, giving an occult metastasis rate of 30.1%. In cases with known or suspected neck metastases, a modified radical neck dissection was typically performed. Similar management was reserved for cases in the CRT group that showed clinical evidence of regional disease 8-12 weeks after primary treatment.

Overall incidence of complications was 20.8% for cases undergoing primary surgery (67/321 cases). Complications mainly included fistula formation, wound healing problems, and bleeding. None of these complications was fatal. A detailed presentation may be found in Table 3. Pharyngeal functional results were satisfactory, as evidenced by the very low rate of permanent gastrostomies (11/321 cases).

**Table 3:** Detailed presentation of complications in cases treated with primary surgery

**Parameter**	**Characteristic**	**Total number (cases)**	**Relative frequency (%)**
**Complications**	*None*	254	79.1
	Bleeding	4	1.2
	Aspiration	6	1.9
	Aspiration pneumonia	1	0.3
	Necrosis of the flap	1	0.3
	Fistula	30	9.3
	General	6	1.9
	Wound healing problems	12	3.7
	Other	5	1.5
	No specifications	2	0.6
	*Total*	321	

## Discussion

The larynx plays a fundamental role in human speech and communication. This fact must always be given consideration when a decision has to be made for the optimal management of a laryngeal tumor. Organ-preservation strategies, either surgical or non-surgical, have dominated the treatment of early laryngeal lesions in recent years [11,12]. A trend toward conservative management has also been noted for locally advanced carcinomas [8,9]. TL is not the only available treatment option for such lesions anymore. Recent developments and newly integrated strategies, including concomitant CRT (CCRT), induction chemotherapy, and modern RT methods have reshaped the field of advanced laryngeal cancer treatment [13-15]. Such a shift in management strategy aims at improved clinical outcome, retention of function, and superior quality of life [9].

Among available organ-preservation modalities, platinum-based CCRT has proven most effective and popular for advanced lesions, showing high rates of laryngeal preservation and satisfactory oncologic results [16,17]. Both radiotherapy and chemotherapy, however, have been associated with severe adverse effects. Such effects locally include dysphagia, xerostomia, trismus, mandibular radionecrosis, fibrosis, and pharyngeal strictures. Systemic adverse effects may also appear and these include bone marrow toxicity, infections, neuropathy, renal failure, nutritional deficiencies, and fatigue. Severe late toxicity has also been recently identified as an important issue associated with CCRT [18]. Additional consideration should be given to the increased incidence of complications following salvage surgery in cases previously treated with CCRT protocols [19].

As combined non-surgical treatment modalities are integrated in the primary management of advanced head and neck cancer it becomes more apparent that organ preservation does not necessarily lead to functional preservation. In other words, simply preserving the larynx does not guarantee its function [20]. Late functional issues following CRT might involve voice as well as swallowing difficulties and in numerous occasions necessitate a permanent tracheostomy and/or gastrostomy. In fact, quality of life in many individuals may end up to be much worse after organ preservation treatment compared to cases that have undergone TL and are able to eat normally and communicate sufficiently with the aid of a prosthesis or other method [21].

Billroth is credited for performing the first TL for cancer in 1873 and for many years this has been the standard of treatment for advanced laryngeal cancer [8,22]. In many areas, however, the application of TL as initial treatment has decreased remarkably [8]. It is now mostly employed as salvage treatment after failure of non-surgical management strategies. Nevertheless, TL may still play an important role as primary therapy for laryngeal cancer. The question whether the most advanced laryngeal lesions with invasion of cartilage are better served with initial non-surgical therapy or TL still remains open. In fact, an advantage in prognosis for surgery in such cases has been previously shown and this remains the main option for management in many areas of the world [23,24]. Moreover, in cases of unreliable patients, or patients who might live in underserved areas, or are not physically fit to undergo the ordeal of CCRT, or even when cost issues are most important, surgery seems to gain the upper hand [8].

In this study, one of the largest T4 laryngeal carcinoma series available in the literature is presented. Between two treatment groups where surgery was primarily applied or not, differences in disease control and survival were noted with the surgical group performing significantly better. As expected, status of the neck on diagnosis and surgical margins also affected survival. A low incidence of complications was generally noted for TL and none of these proved to be fatal. Unfortunately, no comparison between complication rates in the surgical and non-surgical groups could be made as complications in the latter had not been documented.

The data presented here meet many of the limitations inherent in retrospective studies. These limitations include selection bias and use of non-standard treatments with modifications made in radiotherapy and chemotherapy protocols over the years. Moreover, the lack of data regarding complications and functional results for patients managed with non-surgical treatment modalities makes comparison between treatment strategies more difficult. However, the aim of this study was by no means to prove that one type of treatment, i.e. surgery, is superior to other management modalities available today for advanced laryngeal cancer. In fact primary surgery should not be addressed as a single treatment option for T4 laryngeal cancer but rather be included as the initial part of a combined strategy that certainly includes radiotherapy and in many situations chemotherapy as well. In the mind of the authors, comparing complications and functional results between primarily surgical and non-surgical treatment options, although valuable, is of secondary importance here. More important is the motive to provide data supporting a common notion among head and neck surgeons that primary surgery remains a crucial part of T4 laryngeal cancer treatment. This comes in contradiction with another notion that has been recently introduced in the literature pointing that CCRT is a valid option for T4 laryngeal cancer and cartilage invasion should by no means considered a contraindication for enrolment in CCRT protocols [25,26]. If nothing else, it becomes clear that well-designed studies are necessary in order to provide solid evidence regarding the best treatment strategy for advanced laryngeal cancer. In the mean time, and as long as prospective randomized studies are generally lacking, data such as that presented here may prove valuable during treatment decision-making.

## Conclusion

In an era when non-surgical treatment modalities begin to dominate the treatment of advanced laryngeal cancer, this study suggests that surgery remains a key element for successful management of T4 laryngeal lesions. The need for well-designed prospective randomised studies in order to reach safer conclusions is emphasized.

## Competing interests

The authors declare that they have no competing interests.

## Authors’ contributions

AK and GP participated in the design of the study and drafted the manuscript. FW participated in the statistical analysis and helped to draft the manuscript. MK participated in data collection and statistical analysis. JZ made substantial contributions to the final form of the manuscript. GV participated in the design and co-ordination of the study. HI conceived of the study and made substantial contributions in the study design and the final form of the manuscript. All authors read and approved the final manuscript.
